# An Association Test for Ordinal Outcomes in Clustered Data With Informative Cluster Size

**DOI:** 10.1002/pst.70089

**Published:** 2026-03-16

**Authors:** Hasika K. Wickrama Senevirathne, Sandipan Dutta

**Affiliations:** ^1^ Singapore Eye Research Institute Singapore National Eye Centre Singapore Singapore; ^2^ Department of Mathematics and Statistics Old Dominion University Norfolk Virginia USA

**Keywords:** clustered data, cluster‐randomized trial, hypothesis testing, informative cluster size, marginal association, ordinal outcomes

## Abstract

In cluster‐correlated data, the number of observations in a cluster can be associated with the outcome from that cluster. This phenomenon is known as informative cluster size which can occur in cluster‐randomized clinical trial data. Several studies have found that ignoring the issue of informative cluster size can produce biased results in the analysis of clustered data. Most of the existing methods for addressing informative cluster size are suited to continuous outcomes. However, ordinal outcomes and covariates are often encountered in clustered data obtained from large clinical studies. The existing methods for ordinal association testing in clustered data can produce suboptimal results in the presence of informative cluster size. In this article, we propose a new nonparametric method for testing marginal association between ordinal variables in clustered data that can account for informative cluster size. Through simulated data analyses, we show that our new test outperforms the existing alternatives in accurately identifying significant marginal ordinal associations in the presence of informative cluster size. Even if the cluster size is not informative, the performance of our method is comparable to the existing methods. Additionally, we demonstrate the usefulness of our proposed method through an application to a real‐world cluster‐randomized clinical trial data.

## 1 Introduction

Clustered data is a type of correlated data where the observations within a cluster are correlated while observations between different clusters are assumed to be independent. Clustered data are frequently encountered in clinical and biomedical studies, especially in longitudinal studies, dental studies, and cluster‐randomized clinical trials. In such data, the cluster size, that is, the number of units present in a cluster, can vary among different clusters. Moreover, this cluster size can be associated with the outcome from that cluster. This phenomenon is known as informative cluster size [1, 2]. In such scenarios of informative cluster size (ICS), it has been seen that estimators based on averaging over participants, ignoring their cluster identities, may produce substantially different results than cluster‐averaged estimators [2, 3]. As for an example, ICS can occur in the data obtained from multiple clinic visits of cancer patients, where the number of clinic visits (cluster size) of a patient (cluster) can indicate the severity of the disease in that patient. In the context of cluster‐randomized clinical trials where hospitals (clusters) are randomized to treatment arms, larger hospitals may have superior (or inferior) outcomes than smaller hospitals, irrespective of the treatment arm, due to the quality of the staff involved, hospital location, type of patients admitted, number of patients served and many other factors. As a result, it is possible that the outcomes averaged over hospitals may produce different conclusions than the outcomes averaged over patients. This is due to the impact of ICS. This needs to be taken into account for determining the appropriate choice of the average treatment effect [3].

Two pioneer approaches for addressing this ICS in the marginal analysis of outcomes in clustered data were by Hoffman et al. [4] and Williamson et al. [5] Following these, several statistical methods have emerged that implement these two approaches for analyzing varied types of outcomes in clustered data [6, 7, 8, 9, 10]. Moreover, the idea of ICS has been extended to additional layers of informativeness arising from the number of units in a group or a subcluster within a cluster [11, 12, 13].

Most of the methods developed so far, in the context of ICS, involve continuous or numerical outcomes. However, in health studies, non‐continuous categorical outcomes are also frequently encountered. For instance, in a multinational mental health research, DSM‐IV disorder severity was assessed on an ordinal scale (e.g., mild, moderate, and serious) with data clustered by country [14]. Similarly, patient‐reported outcomes in multi‐center oncology trials—such as symptom severity or adverse events—are often recorded using categorical measures, resulting in a clustered ordinal data structure [15]. Other notable examples include the use of the modified Rankin Scale (mRS) to measure functional recovery in multi‐center stroke studies [16] and the grading of diabetic retinopathy severity within multi‐ethnic cohort studies [17]. Despite such prevalence, research on non‐continuous outcomes, in the area of ICS, has been very limited with a few recent exceptions [18, 19]. One of these recent works by Gregg et al. [19] suggests a nonparametric model‐free testing of marginal association between categorical outcomes in clustered data with ICS. Their work is analogous to the Pearson independence test for bivariate categorical outcomes in the context of clustered data with ICS. However, this approach ignores any ordinality that may be present in the categories of the outcome variable. Therefore, the method of Gregg et al. [19] can produce suboptimal inference for association testing of ordinal outcomes, especially when a large observational study or a cluster‐randomized clinical trial produces multiple ordinal outcomes of interest. Selman et al. [20] enlists a large number of recent clinical trials which used ordinal outcomes as primary and secondary endpoints. These include cluster‐randomized clinical trials [21] that generated clustered ordinal outcomes. In such cases of clustered clinical data, the common approach for testing marginal ordinal association turns out to be the classical generalized (stratified) Mantel–Haenszel trend test [22, 23] that treats clusters as strata. Being an ad‐hoc solution for analyzing stratified data, the generalized Mantel–Haenszel trend test is ignorant to the presence of ICS in clustered data. Therefore, an appropriate robust model‐free marginal association testing method for ordinal variables has been missing in the ICS paradigm for clustered data. Motivated by the need to fill this void, in this article, we propose a novel nonparametric test, free of any model‐based assumptions, for testing association between ordinal outcomes in clustered data that can address ICS.

The rest of the article is organized in the following way. In Section 2, we develop our new test for testing marginal association between two ordinal variables in clustered data with an emphasis on ICS. In Section 3, we evaluate the performance of the proposed test in simulated data and compare it with the existing alternative tests. Section 4 demonstrates the applicability and usefulness of our proposed method through real‐world cluster‐randomized clinical trial data involving a diabetes intervention. The article ends with a discussion in Section 5.

## 2 Methodology

### 2.1 Background and Notations

We first discuss a standard mechanism of testing association between two ordinal categorical variables. Based on that, we develop our test statistic of ordinal association testing in a clustered data setting with the primary focus on ICS.

Let X and Y be two ordinal categorical variables having K ordered categories and G ordered categories, respectively. In a classical setting, this categorical data can be represented through a K×G contingency table with the ordered categories of X and Y as rows and columns, respectively. For testing the association between two such ordinal variables in a K×G contingency table, one of the most popular approaches is the Mantel–Haenszel test (MH test) for linear trend as described in Agresti [23], and Mantel and Haenszel [24]. In this approach each category of a variable is assigned a numerical score in such a way that the ordered structure of the categories within a variable is preserved in the scores assigned. Let $u_{1}\leq u_{2}\leq \ldots \leq u_{K}$ denote the assigned scores for the K categories of the X variable and $v_{1}\leq v_{2}\leq \ldots \leq v_{G}$ denote the assigned scores for the G categories of the Y variable. This leads to a two‐way contingency table with K rows and G columns where the cell frequency of a typical cell $\left(k,g\right)$ represents the number of observations having the scores $u_{k}$ and $v_{g}$ for the X and Y variables respectively. The classical MH test uses the correlation coefficient $\left(\rho \right)$ between the two sets of scores for testing whether the two ordinal variables X and Y are associated or not ($H_{0}:\rho =0$). While this approach is known to identify the linear association between the two sets of scores, its interpretation for measuring, or identifying, the strength of association between the two underlying ordinal variables would depend on the choice of the scores used. Since the choice of numerical scores could be arbitrary, e.g., equally spaced versus unequally spaced scores, the accuracy of the results from this approach could depend on the subjective choice of the investigator [24]. One way of circumventing this issue is to use the ranks of the scores, instead of the scores themselves, for both categories. This approach would preserve the ordered nature of the scores and identify any monotonic association while alleviating the problem of subjectivity of the score choices and interpretations. In our method, we use this approach of score selection. One of the main assumptions of the MH test is that all the observations in the data are independent, i.e., every frequency count in each cell of the K×G contingency table is coming from independent sampling units. This assumption of independence becomes invalid in clustered data where observations within a cluster are correlated. Therefore, we need to modify the standard MH test to account for the correlated observations in clustered data. We would formulate the new test statistic based on the following notations.

Suppose there are M clusters in the data. A cluster i has $n_{i++}$ units, i.e., the cluster size is $n_{i++}$ for the cluster i. Let $X_{\mathrm{ij}}$ denote the X‐category and $Y_{\mathrm{ij}}$ denote the Y‐category for a unit j in a cluster i where $1\leq j\leq n_{i++}$ and 1≤i≤M. Then, all the information in the cluster i can be represented through $V_{i}=\left\{n_{i++},X_{\mathrm{ij}},Y_{\mathrm{ij}};1\leq j\leq n_{i++}\right\}$ while $V_{1},V_{2},\ldots ,V_{M}$ are i.i.d. Now, let us consider the two‐way contingency table within each cluster i. Let $n_{\mathrm{ikg}}$ be the number of observations belonging to the row (X‐category) k and the column (Y‐category) g in the cluster i. In other words, the cell frequency for the cell $\left(k,g\right)$ in the cluster i is $n_{\mathrm{ikg}}=\sum_{j=1}^{n_{i++}}I\left(X_{\mathrm{ij}}=k,Y_{\mathrm{ij}}=g)\right)$, for 1≤k<K, 1≤g≤G, with $I\left(.\right)$ being a binary indicator function. Here, the cluster size $n_{i++}=\sum_{k}\sum_{g}n_{\mathrm{ikg}}$. Moreover, in the cluster i, the $k^{\mathrm{th}}$ row frequency is $n_{\mathrm{ik}+}=\sum_{g=1}^{G}n_{\mathrm{ikg}}$ and the $g^{\mathrm{th}}$ column frequency is $n_{i+g}=\sum_{k=1}^{K}n_{\mathrm{ikg}}$. We define ${\hat{\pi }}_{\mathrm{ikg}}=\frac{n_{\mathrm{ikg}}}{n_{i++}}$, ${\hat{\pi }}_{\mathrm{ik}+}=\frac{n_{\mathrm{ik}+}}{n_{i++}}$, and ${\hat{\pi }}_{i+g}=\frac{n_{i+g}}{n_{i++}}$.

### 2.2 Test for Ordinal Association in Clustered Data

#### 2.2.1 Development of the Test Statistic

The classical MH test, discussed in the previous section, would not be applicable for this clustered data as the units within a cluster are not independent. In this case, we can use the idea of within‐cluster‐resampling [4] technique to implement the MH test in this data. Considering there are $n_{i++}$ paired outcomes $\left(X_{\mathrm{ij}},Y_{\mathrm{ij}};1\leq j\leq n_{i++}\right)$ in every cluster i, we randomly select one outcome pair, say $\left(X_{i}^{*},Y_{i}^{*}\right)$, with equal probability $1/n_{i++}$ from the cluster i. This procedure is repeated for each of the M clusters, resulting in a resampled data set $\left\{\left(X_{1}^{*},Y_{1}^{*}\right),\left(X_{2}^{*},Y_{2}^{*}\right),\ldots ,(X_{M}^{*},Y_{M}^{*})\right\}.$ Note that this set of M resampled observations are independent since each of them is obtained from a different cluster. Therefore, based on this set of M independent pairs, we can compute the MH test statistic [23]

$$W^{*}=\sqrt{M-1}\;{\hat{\rho }}^{*}$$

where

$${\hat{\rho }}^{*}=\frac{\sum_{k}\sum_{g}u_{k}v_{g}{\hat{\pi }}_{\mathrm{kg}}^{*}-{\bar{u}}^{*}{\bar{v}}^{*}}{\left(\sqrt{\sum_{k}{u_{k}}^{2}{\hat{\pi }}_{k+}^{*}-{\bar{u}}^{*2}}\right)\left(\sqrt{\sum_{g}{v_{g}}^{2}{\hat{\pi }}_{+g}^{*}-{\bar{v}}^{*2}}\right)}$$

Here, ${\hat{\pi }}_{\mathrm{kg}}^{*}=\frac{n_{\mathrm{kg}}^{*}}{M}$, $n_{\mathrm{kg}}^{*}=\sum_{i=1}^{M}I\left(X_{i}^{*}=k,Y_{i}^{*}=g\right)$, ${\hat{\pi }}_{k+}^{*}=\frac{n_{k+}^{*}}{M}$, ${\hat{\pi }}_{+g}^{*}=\frac{n_{+g}^{*}}{M}$, ${\bar{u}}^{*}=\sum_{k}u_{k}{\hat{\pi }}_{k+}^{*}$, and ${\bar{v}}^{*}=\sum_{g}v_{g}{\hat{\pi }}_{+g}^{*}$.

In the abovementioned MH statistic $W^{*}$, all the M clusters contribute equally by providing one outcome pair each. This alleviates the unequal contributions from differently sized clusters. However, this $W^{*}$, despite being a valid test statistic, is inefficient for testing the association between the ordinal variables for this clustered data. This is because, in the construction of $W^{*}$, only one pair of observation is being considered from the whole cluster and, hence, the value of $W^{*}$ would heavily depend on the pair being selected and subjected to wide sampling fluctuations. To overcome this limitation due to the imposed randomization, we use a marginalization principle [5] where we average the statistic, specifically ${\hat{\rho }}^{*}$, based on resamples across all possible resampled realizations of $\left(X_{i}^{*},Y_{i}^{*}\right)$ values given the observed data. To achieve this for the ratio statistic ${\hat{\rho }}^{*}$, we replace all resampled components in ${\hat{\rho }}^{*}$ with the corresponding averages, or conditional expectations, given the complete clustered data $\left\{V_{1},V_{2},\ldots ,V_{M}\right\}$. Therefore, our proposed new estimator of association between the ordinal variables in clustered data is

$${\hat{\rho }}_{c}=\frac{E\left[\left(\left(\sum_{k}\sum_{g}u_{k}v_{g}{\hat{\pi }}_{\mathrm{kg}}^{*}-{\bar{u}}^{*}{\bar{v}}^{*}\right)|\left\{V_{1},V_{2},\ldots ,V_{M}\right\}\right)\right]}{\sqrt{E\left(\left(\sum_{k}{u_{k}}^{2}{\hat{\pi }}_{k+}^{*}-{\bar{u}}^{*2}\right)|\left\{V_{1},V_{2},\ldots ,V_{M}\right\}\right)}\sqrt{E\left(\left(\sum_{g}{v_{g}}^{2}{\hat{\pi }}_{+g}^{*}-{\bar{v}}^{*2}\right)|\left\{V_{1},V_{2},\ldots ,V_{M}\right\}\right)}}$$

$$=\frac{C_{u,v}}{\sqrt{S_{u}}.\sqrt{S_{v}}}$$

where

$$\begin{array}{c} C_{u,v}=\frac{1}{M}\left(\sum_{i=1}^{M}\sum_{k=1}^{K}\sum_{g=1}^{G}u_{k}v_{g}{\hat{\pi }}_{\mathrm{ikg}}\right)\\ \;-\frac{1}{M^{2}}\left(\left(\sum_{i=1}^{M}\sum_{k=1}^{K}u_{k}{\hat{\pi }}_{\mathrm{ik}+}\right)\left(\sum_{i=1}^{M}\sum_{g=1}^{G}v_{g}{\hat{\pi }}_{i+g}\right)\right) \end{array}$$

$$\begin{array}{c} S_{u}=\frac{M-1}{M^{2}}\left(\sum_{i=1}^{M}\sum_{k=1}^{K}{u_{k}}^{2}{\hat{\pi }}_{\mathrm{ik}+}\right)+\frac{1}{M^{2}}\left(\sum_{i=1}^{M}\sum_{k=1}^{K}\sum_{g=1}^{G}u_{k}^{2}{{\hat{\pi }}_{\mathrm{ikg}}}^{2}\right)\\ \;-{\left(\frac{1}{M}\sum_{i=1}^{M}\sum_{k=1}^{K}u_{k}{\hat{\pi }}_{\mathrm{ik}+}\right)}^{2} \end{array}$$

$$\begin{array}{c} S_{v}=\frac{M-1}{M^{2}}\left(\sum_{i=1}^{M}\sum_{g=1}^{G}v_{g}^{2}{\hat{\pi }}_{i+g}\right)+\frac{1}{M^{2}}\left(\sum_{i=1}^{M}\sum_{k=1}^{K}\sum_{g=1}^{G}v_{g}^{2}{{\hat{\pi }}_{\mathrm{ikg}}}^{2}\right)\\ \;-{\left(\frac{1}{M}\sum_{i=1}^{M}\sum_{g=1}^{G}v_{g}{\hat{\pi }}_{i+g}\right)}^{2}. \end{array}$$

The detailed calculations for obtaining the above expression of ${\hat{\rho }}_{c}$ can be found in Appendix A.

Then, our proposed test statistic, extending the idea of the MH test, for testing ordinal association in clustered data is $W_{c}=\sqrt{M-1\;}{\hat{\rho }}_{c}$.

#### 2.2.2 Variance Estimation and Testing Procedure

The classical MH test statistic, $W^{*}$, is supposed to have an asymptotic standard normal distribution, for large M, under the null hypothesis, $H_{0}$, of no association between the ordinal variables [23]. Asymptotic normality of the statistics based on the within‐cluster resampling and the equivalent marginalization procedures have also been established in previous research works [4, 5]. While our proposed estimator, ${\hat{\rho }}_{c}$, can be shown to be asymptotically (for large M) unbiased under $H_{0}$ (see the Supplementary Web Material for an empirical justification), to implement a large sample testing, similar to the aforementioned tests, we need to estimate the variance of our proposed test statistic $W_{c}$. To obtain this variance estimate, $\hat{V}\left(W_{c}\right)$, we employ a ‘delete‐1‐cluster’ jackknife‐based variance estimation [12].

Let $W_{c\left(i\right)}$ be the value of the test statistic obtained after deleting the $i^{\mathrm{th}}$ cluster. In other words, 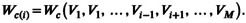 The jackknife variance estimate of $W_{c}$ is

$$\hat{V}\left(W_{c}\right)=\frac{M-1}{M}\sum_{i=1}^{M}{\left(W_{c\left(i\right)}-W_{c\left(.\right)}\right)}^{2},$$

where

$$W_{c\left(.\right)}=\frac{\sum_{i=1}^{M}W_{c\left(i\right)}}{M}.$$

Then, the standardized test statistic $Z_{c}=W_{c}/\sqrt{\hat{V}\left(W_{c}\right)}$, which would follow a standard normal distribution for large M under $H_{0}$, can be used to carry out the test of association between the two ordinal variables in a clustered data. The empirical performance of this proposed test has been evaluated in the next section.

## 3 Simulation Studies

In this section, we evaluate the performance of the proposed test through empirical type‐I error rate and power calculations in different simulated settings of clustered data. We also compare it with two existing alternative nonparametric tests for clustered categorical data. One of the tests is the generalized Mantel–Haenszel (GMH) test [22] which uses clusters as strata while carrying out the MH test. The other test is the association test by Gregg, Datta, and Lorenz [19] developed for clustered categorical data which would be referred to as the GDL test. Note that, while GDL test can be implemented with different choices for the variance estimator, we have used the method of moments version of the GDL test following the recommendation in the original article [19]. We considered a variety of simulation settings that varies in terms of the number of X‐categories and Y‐categories, i.e., K and G. In each setting, we consider three different choices of the number of clusters (M), namely, 25, 50, and 100 representing small, moderate, and large sample scenarios, respectively. The chosen nominal (target) size or type‐I error rate is 0.05. The empirical size (type‐I error rate), along with its 95% confidence interval, and the power results, are based on 2000 Monte‐Carlo iterations.

### 3.1 Clustered Data With ICS

In this simulation setting, we have two ordinal categorical variables (X and Y) measured in each of the M clusters. In a cluster i, 1≤i≤M, two independent variables $a_{i}$ and $q_{i}$ are generated where $a_{i}\sim \text{Normal}\left(0,1\right)$ and $q_{i}\sim \text{Normal}\left(0,0.01\right)$. For the same cluster, the cluster size $n_{i++}$ is generated from a Poisson distribution, $\left(n_{i++}-1\right)\sim \text{Poisson}\left(20\left(\exp\left(0.5a_{i}q_{i}\right)\right)\right).$ We generate $X_{\mathrm{ij}}$, the ordinal category score of the X variable for the unit j of cluster i, by discretizing $Q_{\mathrm{ij}}=a_{i}+e_{\mathrm{ij}}^{X}$ into one of the K ordered categories. Here, $e_{\mathrm{ij}}^{X}\sim \text{Normal}\left(0.1,1\right)$ independently for all $j=1,2,3\ldots ,n_{i++}$. In the discretization procedure, the cut points defining the K ordered categories of the X variable are based on the empirical equal‐probability quantiles that ensure balanced frequencies across all the categories. Here, $X_{\mathrm{ij}}$ is assigned a value from $\left\{1,2,3\ldots ,K\right\}$ equivalent to the rank of the X‐category. Then, we generate $Y_{\mathrm{ij}}$, the ordinal category score of the Y variable for the unit j of cluster i. This is done, similar to $X_{\mathrm{ij}}$, by the discretization of $P_{\mathrm{ij}}=q_{i}+e_{\mathrm{ij}}^{Y}+\delta X_{\mathrm{ij}}$ into one of the G ordered categories through empirical equal‐probability quantiles. Here, $e_{\mathrm{ij}}^{Y}\sim \text{Normal}\left(0,2\right)$ independently for all j and δ is the parameter controlling the degree of association between the two ordinal variables X and Y. In case of δ=0, we have no association between the two ordinal variables which represent our null hypothesis. On the other hand, with an increase in the absolute value of δ, the strength of the association between the two variables increases. In this simulation, we have considered different choices of δ. Also, $Y_{\mathrm{ij}}$ is assigned a value from $\left\{1,2,3\ldots ,G\right\}$ equivalent to the rank of the Y‐category. In this way, we generate $n_{i++}$ pairs of $\left(X_{\mathrm{ij}},Y_{\mathrm{ij}}\right)$ in each cluster i where 1≤i≤M. Note that the cluster size $n_{i++}$, in a cluster i, is correlated with the variables X and Y through the random cluster effects $a_{i}$ and $q_{i}$, respectively. This results in a clustered ordinal data with informative cluster size.

In this simulated setting, we have considered different choices for the number of clusters $\left(M\right)$, namely, 25, 50, and 100, which would represent small, moderate, and large sample scenarios, respectively. For each of the three choices of M, we have considered different choices for the number of X and Y categories, i.e., K and G. In this section we present the results in terms of K×G contingency tables by choosing values of both K and G from the set $\left\{2,3,4\right\}$. This allows us to have a variety of contingency table settings that include equal as well as unequal number of categories in the two ordinal variables. In each of these simulated settings, we obtain the empirical size or type‐I error rate and the empirical power of our proposed test along with that of the competing GMH and GDL tests. The empirical type‐I error rate of a test is calculated as the proportion of rejections (p‐value < 0.05) in the Monte‐Carlo iterations when the data is generated under the null hypothesis model, i.e., δ=0. The empirical power of a test is obtained as the proportion of rejections, in the Monte‐Carlo iterations, for a value of δ≠0, i.e., under the alternative hypothesis. The results from this simulation setting are presented as two different cases as follows.

Case 1 At least one of the two ordinal variables is binary.

Here, we consider the situation where the value of K is 2, i.e., the variable X is binary. The other variable can have two or more ordered categories, i.e., G can have any value from the set $\left\{2,3,4\right\}$. In these combinations, the ordering of the two categories of the binary variable X is not expected to have any additional significant impact on the testing of association or independence. Additionally, this set of choices for the two‐way contingency table would include the 2×2 scenario where both variables have only two categories and they can be essentially treated as two nominal categorical variables disregarding their ordinality. Hence, this setting is in line with the GDL test since GDL test assumes the variables to be nominal categorical variables without considering any specific ordering of its categories.

**FIGURE 1:**
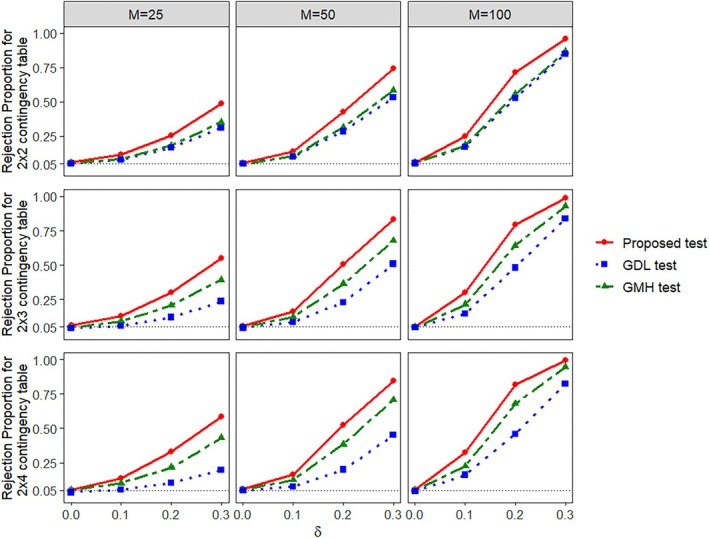
Empirical power (rejection proportion) of the proposed test, GMH test, and GDL test from Section 3.1 when K=2 and G≥2 in clustered data with ICS.

Table 1 shows the empirical size (type‐I error rate), along with its 95% confidence interval, for all three tests under study for different choices of M and K×G combinations. From this table, we find that the empirical size of the proposed test is close to the nominal size of 0.05 for the different combinations of K×G in small, moderate, and large number of clusters. The GMH test is also consistent in maintaining the nominal size of 0.05. On the other hand, the GDL test tends to be more conservative than the other two tests, especially for the small M and G>2. Figure 1 shows the empirical power performances of all the three tests. The empirical power curve is computed as a function of the association strength measure δ. Power values of all the three tests increase with increase in the values of δ and M. From this figure, we see that the power of our proposed test, for any δ≠0, is always the highest among the three tests for all the choices of M and G. The GDL test has the lowest power in all the scenarios apart from the 2×2 scenarios where the power performances the GMH test and the GDL test are similar. Overall, in this setting, our proposed test is the one with the highest power while maintaining the nominal type‐I error rate.

Case 2 Both ordinal variables have more than two categories.

**TABLE 1:** Empirical size (95% confidence interval) of the proposed test, GMH test, and GDL test from Section 3.1 when K = 2 and G≥2 in clustered data with ICS.

M	K×G	Proposed test	GMH test	GDL test
25	2×2	0.060 (0.050, 0.070)	0.056 (0.046, 0.066)	0.050 (0.040, 0.059)
	2×3	0.061 (0.051, 0.071)	0.045 (0.036, 0.054)	0.041 (0.032, 0.050)
	2×4	0.055 (0.045, 0.065)	0.050 (0.040, 0.060)	0.035 (0.027, 0.043)
50	2×2	0.053 (0.043, 0.063)	0.044 (0.035, 0.052)	0.049 (0.039, 0.058)
	2×3	0.058 (0.048, 0.068)	0.049 (0.040, 0.058)	0.044 (0.035, 0.052)
	2×4	0.058 (0.048, 0.068)	0.052 (0.042, 0.062)	0.049 (0.039, 0.058)
100	2×2	0.058 (0.048, 0.068)	0.058 (0.048, 0.068)	0.053 (0.043, 0.062)
	2×3	0.051 (0.041, 0.061)	0.049 (0.040, 0.058)	0.048 (0.039, 0.057)
	2×4	0.052 (0.042, 0.062)	0.050 (0.040, 0.060)	0.044 (0.035, 0.053)

**FIGURE 2:**
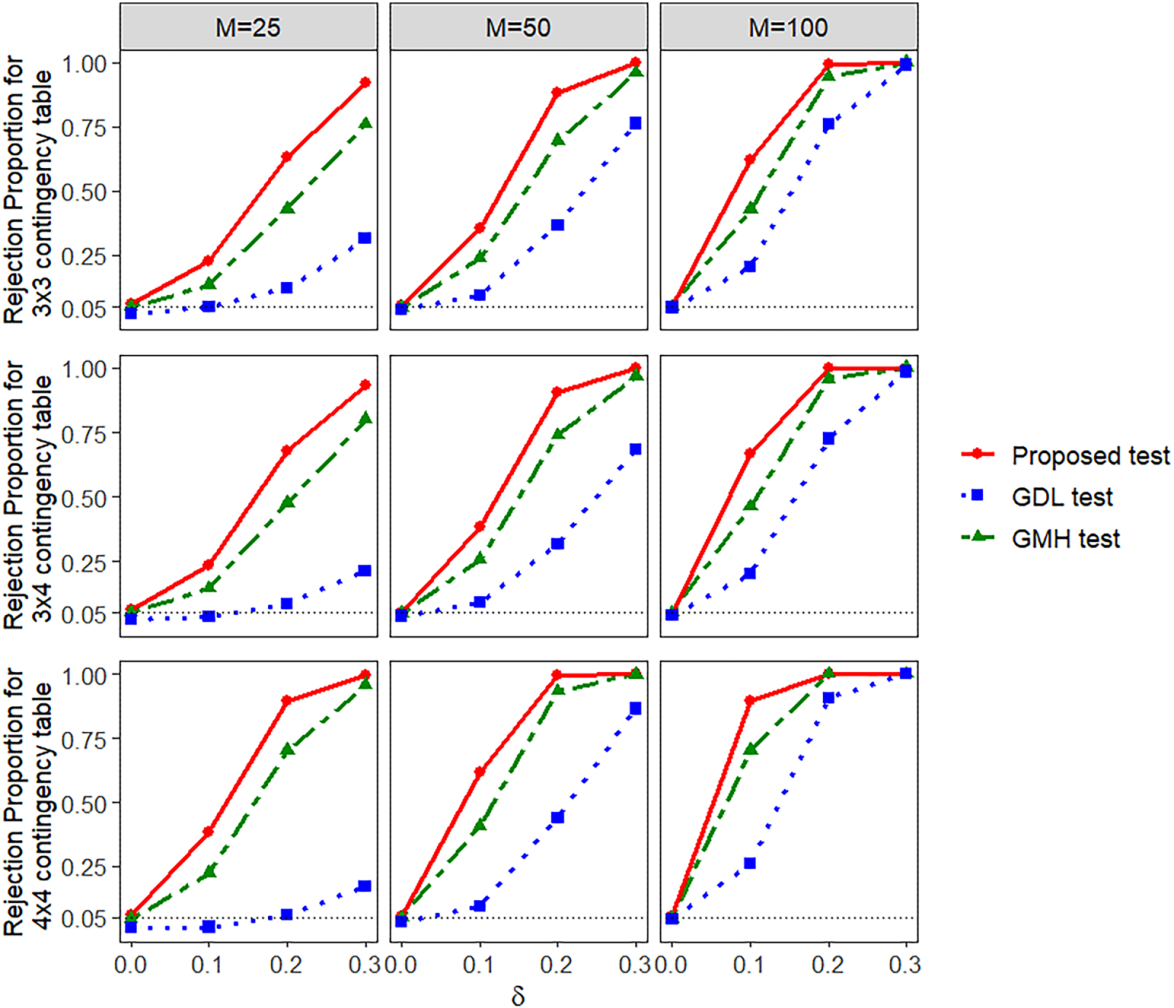
Empirical power (rejection proportion) of the proposed test, GMH test, and GDL test from Section 3.1 when K>2 and G>2 in clustered data with ICS.

Here, we consider the results of the simulation scenarios consisting of 3×3, 3×4, and 4×4 contingency tables. Table 2 shows the empirical size (type‐I error rate), and the corresponding 95% confidence interval, of all three tests under study for different choices of M. From this table, we find that our proposed test closely maintains the target type‐I error rate for different numbers of clusters and contingency table configurations. The same can be said about the GMH test. However, the GDL test seems overly conservative, particularly when dealing with a small or moderate number of clusters and many ordinal categories. Figure 2 shows the empirical power performances of the three tests for different number of clusters and contingency table settings. The power values of all the three tests increase with the increase in the number of clusters as well as with an increasing δ. However, our proposed test dominates the other two tests, in terms of power, with its power values being higher than the other two in all the three contingency table settings for any given choice of $\delta \left(\neq 0\right)$ and M. The GDL test has the lowest power values among the three tests in all the scenarios with its power being extremely low for the setting M=25. This highlights that GDL test may not be a suitable candidate in case of a small number of clusters, especially for M<30, and it needs a larger M than the other two tests to reach its asymptotic power.

**TABLE 2:** Empirical size (95% confidence interval) of the proposed test, GMH test, and GDL test from Section 3.1 when K>2 and G>2 in clustered data with ICS.

M	K×G	Proposed test	GMH test	GDL test
25	3×3	0.061 (0.051, 0.071)	0.050 (0.040, 0.060)	0.024 (0.017, 0.031)
	3×4	0.062 (0.051, 0.072)	0.051 (0.041, 0.061)	0.024 (0.017, 0.031)
	4×4	0.061 (0.051, 0.071)	0.045 (0.036, 0.054)	0.009 (0.005, 0.013)
50	3×3	0.053 (0.043, 0.063)	0.047 (0.038, 0.056)	0.041 (0.032, 0.050)
	3×4	0.051 (0.041, 0.061)	0.048 (0.039, 0.057)	0.038 (0.030, 0.046)
	4×4	0.054 (0.044, 0.064)	0.049 (0.040, 0.058)	0.033 (0.025, 0.041)
100	3×3	0.049 (0.040, 0.058)	0.050 (0.040, 0.060)	0.047 (0.038, 0.056)
	3×4	0.046 (0.036, 0.055)	0.051 (0.041, 0.061)	0.040 (0.031, 0.049)
	4×4	0.057 (0.047, 0.067)	0.047 (0.038, 0.056)	0.042 (0.033, 0.051)

In conclusion, through these simulations we find that our proposed test is the best performing method maintaining the nominal type‐I error rate while producing higher power than the existing alternative tests for identifying association between ordinal variables in a clustered data with informative cluster size.

### 3.2 Clustered Data Without ICS

For this simulation setting we generate the cluster size $n_{i++}$, in a cluster i, in a different way from the previous simulation setting 3.1 so that there are no impacts of the random cluster effects $a_{i}$ and $q_{i}$ on the cluster size. Specifically, for 1≤i≤M, $\left(n_{i++}-1\right)\sim \text{Poisson}\left(20\left(\exp\left(0.5\right)\right)\right)$ while the two sets of ordinal scores, $X_{\mathrm{ij}}$ and $Y_{\mathrm{ij}}$ ($1\leq j\leq n_{i++}$), are generated in the same way as in the previous simulation setting 3.1. Since the random cluster effects, $a_{i}$ and $q_{i}$, were the only sources of association between the cluster size and the ordinal categorical variables in the previous simulation setting, generating $n_{i++}$ independent of $a_{i}$ and $q_{i}$ made the outcome variables independent of the cluster size in a cluster i. Hence, the clustered data generated in this simulation setting is devoid of ICS.

**FIGURE 3:**
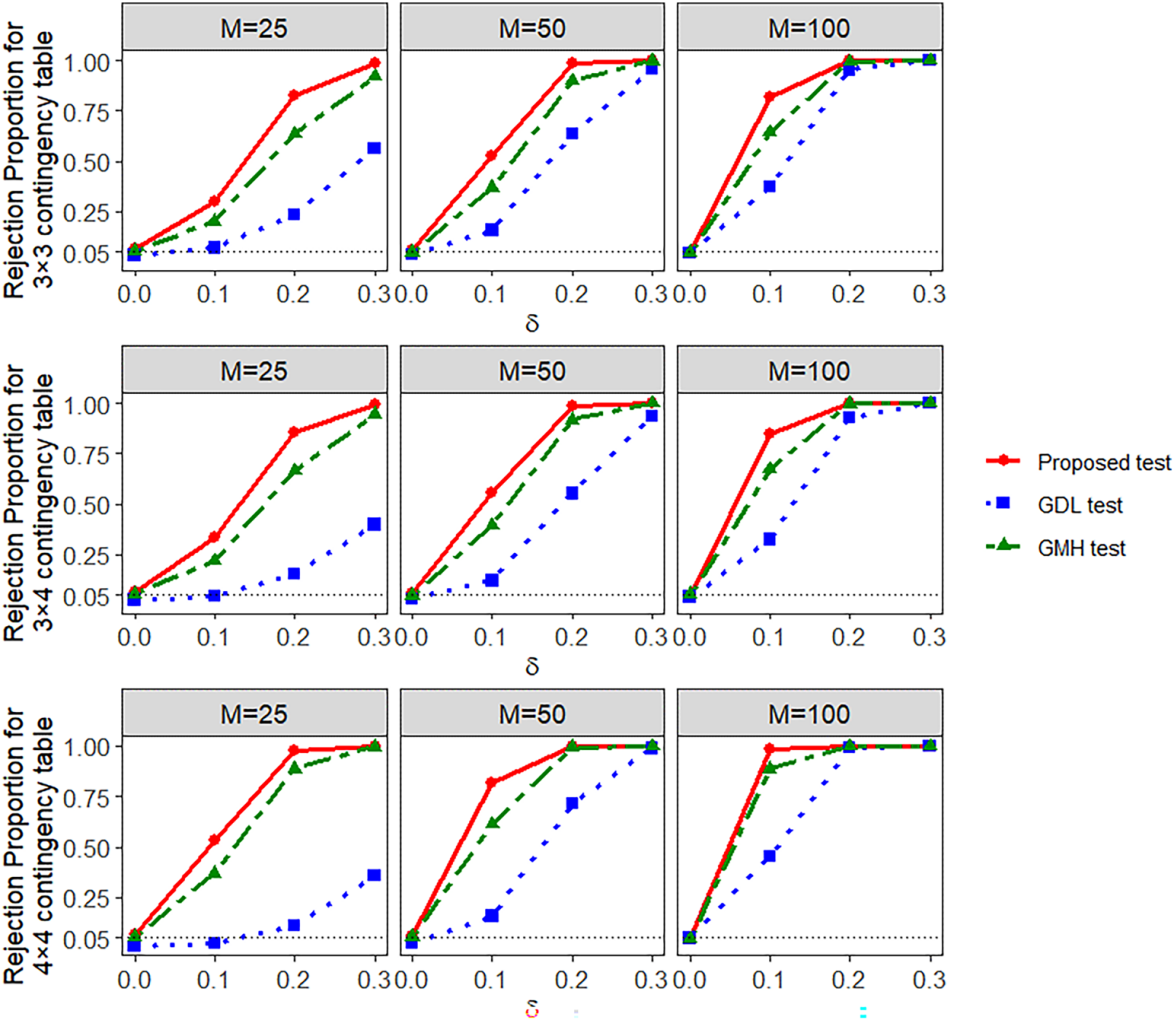
Empirical power (rejection proportion) of the proposed test, GMH test, and GDL test from Section 3.2 when K>2 and G>2 in clustered data without ICS.

Table 3 shows the empirical size (type‐I error rate), with its 95% confidence interval, of the proposed test as well as that of the GDL test and the GMH test in clustered data without any ICS. We see that our proposed test and the GMH test have comparable size values, although the proposed test appears to be more liberal at M=25 compared to the higher values of M. On the other hand, the GDL test continues its pattern of conservative performance with its empirical size consistently remaining way below the target 0.050 mark, especially for M=25 and 50. This is further reflected in Figure 3 where we compare the power performances of the three tests for different choices of δ. The GDL, unsurprisingly, has the lowest power out of the three, while our proposed test has the highest power values, for all choices of the effect size δ in all the combinations of K×G and different number of clusters (M). While the power of the GMH test is still less than that of our proposed test even in the absence of ICS, the differences between the two sets of power values appear to have reduced in case of large samples (M=100) when compared to the results of the clustered data with ICS. This difference, however, continues to be substantial in small and moderate samples.

**TABLE 3:** Empirical size (95% confidence interval) of the proposed test, GMH test, and GDL test from Section 3.2 when K>2 and G>2 in clustered data without ICS.

M	K×G	Proposed test	GMH test	GDL test
25	3×3	0.064 (0.053, 0.075)	0.054 (0.044, 0.063)	0.034 (0.026, 0.042)
	3×4	0.065 (0.054, 0.075)	0.056 (0.045, 0.066)	0.026 (0.019, 0.033)
	4×4	0.068 (0.057, 0.078)	0.054 (0.044, 0.063)	0.009 (0.005, 0.013)
50	3×3	0.059 (0.048, 0.069)	0.047 (0.038, 0.056)	0.036 (0.028, 0.044)
	3×4	0.062 (0.051, 0.073)	0.047 (0.038, 0.056)	0.029 (0.022, 0.036)
	4×4	0.060 (0.049, 0.070)	0.052 (0.042, 0.061)	0.024 (0.017, 0.031)
100	3×3	0.055 (0.045, 0.065)	0.048 (0.038, 0.057)	0.046 (0.037, 0.055)
	3×4	0.051 (0.041, 0.061)	0.053 (0.043, 0.062)	0.041 (0.032, 0.050)
	4×4	0.051 (0.041, 0.061)	0.044 (0.035, 0.052)	0.048 (0.039, 0.057)

Therefore, in conclusion, our proposed test is the best performing method even if the cluster sizes are not informative and, hence, can be trustfully used for ordinal association testing in any clustered data.

## 4 Real Data Analysis

In this section, we illustrate the application of our proposed test using a real‐world cluster‐randomized clinical trial data. The data analyzed in this section arises from the Kerala Diabetes Prevention Program (K‐DPP) [25]. This is a lifestyle‐intervention program that aimed to prevent the disease progression of, or reduce the degree of morbidity associated with, Type‐2 diabetes mellitus (T2DM) in an at‐risk population in a populated district of the state of Kerala in India. This study was adapted to a rural India population with the goal of benefiting from lifestyle intervention cures in a low‐income or middle‐income population similar to that achieved in the evidence‐based peer‐driven lifestyle intervention programs in high‐income countries like Australia, Finland, and United States. In the K‐DPP trial, the primary outcome was diabetes status or incidence, while several other secondary outcomes or variables including, clinical or biochemical outcomes (e.g., body‐mass index, blood pressure, cholesterol levels etc.), behavioral variables (e.g., alcohol consumption, tobacco usage, physical work), and sociodemographic measures (e.g., education level, occupation) were also considered. In this trial, 60 polling areas or subdivisions were randomized into the two arms, control and intervention, under equal allocation ratio. This resulted in 30 subdivisions, having 507 participants, being randomized to the control arm while the other 30 subdivisions, with 500 participants, receiving the intervention. Therefore, the subdivisions were treated as clusters with 30 clusters in each arm of the trial. Data was collected at baseline, 12 months, and 24 months. In our analysis, we focus on studying the association of the primary diabetes outcome with two other ordinal variables, namely, BMI category and physical work status.

**FIGURE 4:**
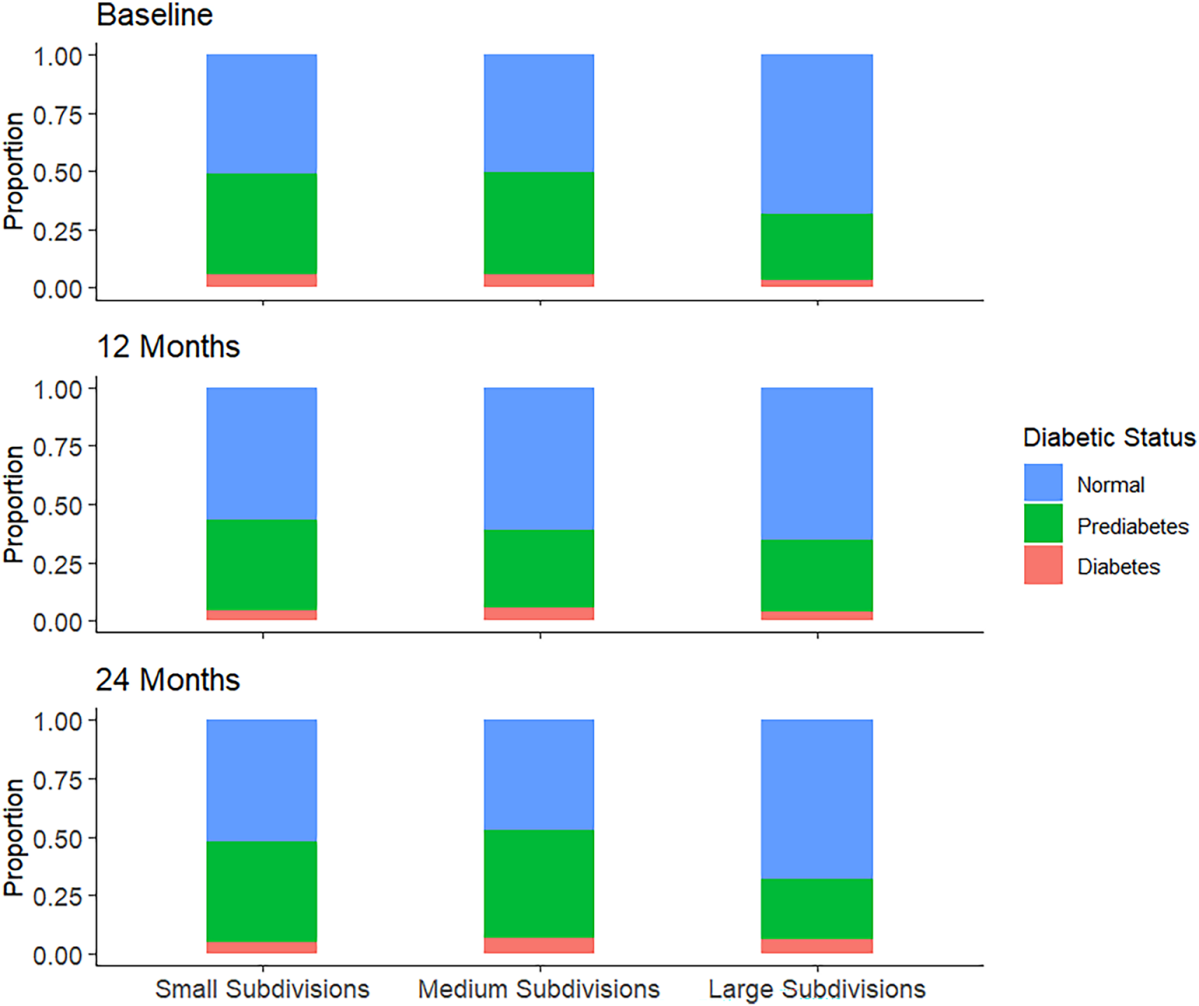
Figure showing the distributions of diabetes status categories by subdivision sizes at different time points of the K‐DPP trial.

Being a cluster‐randomized trial, all the participants belonging to a given cluster (subdivision) are placed in the same arm of the trial. However, the number of participants in a cluster, i.e., the cluster size, varied from one cluster to another ranging from 9 to 24. Therefore, clusters with more participants, i.e., larger subdivisions, contribute more data points to the marginal analysis than smaller clusters or subdivisions. In this case, if an outcome, say, diabetes incidence, is correlated to the cluster size, then there would be an imbalance or bias in the contributions between the diabetic population and the non‐diabetic populations. This is where the cluster size becomes informative, i.e., we can have ICS in the clustered data. This is a possible scenario in the K‐DPP trial data since the quality of lifestyle and healthcare vary widely among the different subdivisions (clusters) of a state. An indication of ICS in this trial can also be seen in Figure 4. We have used the primary outcome of diabetes status which has three ordinal categories (levels): “Normal,” “Prediabetes,” and “Diabetes,” based on the American Diabetes Association (ADA) classification. In Figure 4, we have categorized the 60 subdivisions into three categories of “small,” “medium,” and “large” subdivisions, using the tertiles of the cluster (subdivision) sizes. It can be observed from the stacked bar charts in Figure 4 that the proportion of individuals having diabetes or prediabetes tend to be decreasing with the increase in the subdivision size. In other words, the smaller subdivisions tend to have higher proportions of diabetes and prediabetes than the larger subdivisions. This pattern is consistent in all the three time points (baseline, 12 months, and 24 months) of the study. Hence, we can suspect that the clustered data generated from the K‐DPP trial has a potential ICS scenario. This feature needs to be taken into account when testing association between diabetes status and other important ordinal variables.

We apply our proposed methodology, that can account for ICS, for testing association of diabetes status and different ordinal outcomes mentioned before. We also apply the traditional GMH test for ordinal association testing to check the extent to which both methods agree or disagree in their results. In all the analyses, a *p*‐value less than 0.050 was assumed to be significant while a *p*‐value between 0.050 and 0.100 was considered as marginally significant. We have tested the associations, based on the 60 clusters combining the two arms of the trial. We test these associations at baseline and at the last follow‐up (24 months). The diabetes status has three ordered categories of “Normal” or non‐diabetes, “Prediabetes,” and “Diabetes.” The body‐mass index or BMI categories are normal, overweight, and obese. The physical work status involves two categories, namely, vigorous and non‐vigorous work. The results of the association testing are given in Table 4. From the diabetes‐BMI association testing at baseline, the GMH test shows marginally significant association while our proposed test does not show any significant association. However, at 24‐months, both GMH and the proposed test identify significant association between diabetes status and BMI levels. At this point, positive values of both test statistics indicate that higher BMI levels tend to be associated with worse diabetes outcomes. From the association testing results of diabetes and vigorous physical activity status, in Table 4, we find that, at baseline, the new test shows marginally significant association between the two ordinal variables, while the GMH test does not show any form of significance. On the other hand, from the 24‐months trial data, our test identifies significant association between diabetes status and physical activity while the GMH shows marginal significance. Based on the 24‐month data, it appears that vigorous physical work is associated with better (lower) diabetes outcomes as indicated through the negative test statistic values for both tests.

**TABLE 4:** Table showing the results of the proposed test and the GMH test in association testing of diabetes status with other ordinal variables in the K‐DPP data.

Variable	Trial phase	*p* (test statistic)	
		Proposed test	GMH test
BMI	Baseline	0.665 (0.117)	0.073 (1.795)
	24‐month	< 0.001 (0.956)	0.002 (3.089)
Vigorous work	Baseline	0.053 (−0.486)	0.745 (−0.326)
	24‐month	0.045 (−0.560)	0.064 (−1.854)

## 5 Discussion

In this article, we have proposed a new nonparametric test for testing association between two ordinal variables in clustered data. Our test can address the complex issue of informative cluster size (ICS) where the outcome from a cluster is correlated with its cluster size. None of the existing nonparametric tests for marginal ordinal association account for ICS, resulting in biased or suboptimal inference in clustered data as shown in our simulation studies. Our test outperforms the classical marginal ordinal association test as well as the existing test for the association of categorical (nominal) outcomes in clustered data with ICS. Even if the cluster size is not informative, our proposed test has been shown to be competitive with the existing tests in terms of accurately detecting ordinal association. We have also demonstrated a real‐life application of our proposed test using cluster‐randomized trial data for a diabetes intervention.

In the construction of the new test, we have applied the idea of identifying monotonic ordinal associations through rank‐based correlations. An alternative approach of identifying association between ordinal variables could be based on Kendall's $\tau_{b}$ measure. A comparison between the tests implementing the rank‐based measure and the Kendall's $\tau_{b}$ measure would be an interesting future project in the context of clustered ordinal association testing under ICS.

In some clustered data, there could be another level of informativeness, in addition to ICS, due to the presence of multiple groups of units within the same cluster. In that case, the number of units belonging to a group in a cluster can be correlated with the outcome from that group in that cluster. This scenario is known as the informative intra‐cluster group size [12, 26]. We plan to extend our ordinal testing mechanism for ICS to clustered data with informative intra‐cluster group size in a future project.

In this article, we introduced a new nonparametric test of association between ordinal variables in clustered data that accounts ICS while remaining robust and model‐free. However, if a researcher's aim extends beyond marginal ordinal association to include modeling an ordinal outcome in clustered data with ICS while adjusting for other continuous or non‐categorical covariates (or their effects), the method of Mitani et al. [18], which is based on a proportional odds model assumption, is an appropriate choice. While our current article is limited to testing only the marginal association between ordinal variables, in future work, we plan to extend this procedure to incorporate the effects of other continuous and non‐categorical covariates, potentially via a model‐based approach comparable to the Mitani et al. [18] method.

## Funding

The authors have nothing to report.

## Conflicts of Interest

The authors declare no conflicts of interest.

## Supporting information


**Table S1:** Average bias (Monte‐Carlo based) of the proposed ordinal association estimator ${\hat{\rho }}_{c}$, under true H0 of no association, for different choices for 𝑀 and the combinations of 𝐾×𝐺 contingency table under the simulation from Section 3.1.
**Table S2:** Average jackknife estimate of the bias of the proposed ordinal association estimator ${\hat{\rho }}_{c}$, under true H0 of no association, for different choices for 𝑀 and the combinations of 𝐾×𝐺 contingency table under the simulation from Section 3.1.

## Data Availability

The Kerala Diabetes Prevention Program (K‐DPP) trial data that has been used in this article is available at https://figshare.com/articles/dataset/K‐DPP_datasets/5661610.

## Appendix A

Detailed calculations (under $H_{0}$) for obtaining the expression of ${\hat{\rho }}_{c}$ in Section 2.2.1.

(A1)
$$\text{Note},{\hat{\rho }}_{c}=\frac{E\left[\left(\sum_{k}\sum_{g}u_{k}v_{g}{\hat{\pi }}_{\mathrm{kg}}^{*}-{\bar{u}}^{*}{\bar{v}}^{*}\right)|\left\{V_{1},V_{2},\ldots ,V_{M}\right\}\right]}{\sqrt{E\left[(\sum_{k}{u_{k}}^{2}{\hat{\pi }}_{k+}^{*}-{\bar{u}}^{*2}|\left\{V_{1},V_{2},\ldots ,V_{M}\right\}\right]}\;\sqrt{E\left[(\sum_{g}{v_{g}}^{2}{\hat{\pi }}_{+g}^{*}-{\bar{v}}^{*2}|\left\{V_{1},V_{2},\ldots ,V_{M}\right\}\right]}}$$

Now we evaluate the different components of the numerator of ${\hat{\rho }}_{c}$ in equation (A1) as follows.

$$E\left[\left(\sum_{k}\sum_{g}u_{k}v_{g}{\hat{\pi }}_{\mathrm{kg}}^{*}\right)|\left\{V_{1},V_{2},\ldots ,V_{M}\right\}\right]=\sum_{k}\sum_{g}u_{k}v_{g}{\hat{\pi }}_{\mathrm{kg}}^{*}\;E\left[\left({\hat{\pi }}_{\mathrm{kg}}^{*}\right)|\left\{V_{1},V_{2},\ldots ,V_{M}\right\}\right]$$

$$=\sum_{k}\sum_{g}u_{k}v_{g}\;E\left[\left(\frac{n_{\mathrm{kg}}^{*}}{M}\right)|\left\{V_{1},V_{2},\ldots ,V_{M}\right\}\right]$$

$$=\frac{1}{M}\sum_{k}\sum_{g}u_{k}v_{g}\;E\left[\sum_{i}I\left(X_{i}^{*}=k,Y_{i}^{*}=g\right)|\left\{V_{1},V_{2},\ldots ,V_{M}\right\}\right]$$

(A2)
$$=\frac{1}{M}\sum_{i}\sum_{k}\sum_{g}u_{k}v_{g}{\hat{\pi }}_{\mathrm{ikg}}$$

$$E\left[\left({\bar{u}}^{*}{\bar{v}}^{*}\right)|\left\{V_{1},V_{2},\ldots ,V_{M}\right\}\right]=E\left[\sum_{k}u_{k}{\hat{\pi }}_{k+}^{*}\sum_{g}v_{g}{\hat{\pi }}_{+g}^{*}|\left\{V_{1},V_{2},\ldots ,V_{M}\right\}\right]$$

$$=E\left[\sum_{k}u_{k}{\hat{\pi }}_{k+}^{*}|\left\{V_{1},V_{2},\ldots ,V_{M}\right\}\right]E\left[\sum_{g}v_{g}{\hat{\pi }}_{+g}^{*}|\left\{V_{1},V_{2},\ldots ,V_{M}\right\}\right]$$

$$=\sum_{k}\sum_{g}u_{k}E\left[{\hat{\pi }}_{\mathrm{kg}}^{*}|\left\{V_{1},V_{2},\ldots ,V_{M}\right\}\right]\sum_{k}\sum_{g}v_{g}E\left[{\hat{\pi }}_{\mathrm{kg}}^{*}|\left\{V_{1},V_{2},\ldots ,V_{M.}\right\}\right]$$

$$=\sum_{k}\sum_{g}u_{k}E\left[\left(\frac{n_{\mathrm{kg}}^{*}}{M}\right)|\left\{V_{1},V_{2},\ldots ,V_{M}\right\}\right]\sum_{k}\sum_{g}v_{g}E\left[\left(\frac{n_{\mathrm{kg}}^{*}}{M}\right)|\left\{V_{1},V_{2},\ldots ,V_{M}\right\}\right]$$

$$\begin{array}{c} =\left(\frac{1}{M}\right)\sum_{k}\sum_{g}u_{k}E\left[\sum_{i}I\left(X_{i}^{*}=k,Y_{i}^{*}=g\right)|\left\{V_{1},V_{2},\ldots ,V_{M}\right\}\right]\\ \times \left(\frac{1}{M}\right)\sum_{k}\sum_{g}v_{g}E\left[\sum_{i}I\left(X_{i}^{*}=k,Y_{i}^{*}=g\right)|\left\{V_{1},V_{2},\ldots ,V_{M}\right\}\right] \end{array}$$

$$=\left(\frac{1}{M^{2}}\right)\left[\sum_{i}\sum_{k}\sum_{g}u_{k}{\hat{\pi }}_{\mathrm{ikg}}\right]\left[\;\sum_{i}\sum_{k}\sum_{g}v_{g}{\hat{\pi }}_{\mathrm{ikg}}\right]$$

(A3)
$$=\left(\frac{1}{M^{2}}\right)\left[\sum_{i}\sum_{k}u_{k}{\hat{\pi }}_{\mathrm{ik}+}\right]\left[\sum_{i}\sum_{g}v_{g}{\hat{\pi }}_{i+g}\right]$$

Next, we evaluate the different components of the denominator of ${\hat{\rho }}_{c}$ in equation (A1) as follows.

(A4)
$$\begin{array}{c} \sqrt{E\left[\left(\sum_{k}{u_{k}}^{2}{\hat{\pi }}_{k+}^{*}-{\bar{u}}^{*2}\right)|\left\{V_{1},V_{2},\ldots ,V_{M}\right\}\right]}\\ =\sqrt{E\left[\sum_{k}{u_{k}}^{2}{\hat{\pi }}_{k+}^{*}|\left\{V_{1},V_{2},\ldots ,V_{M}\right\}\right]-E\left[{\bar{u}}^{*2}|\left\{V_{1},V_{2},\ldots ,V_{M}\right\}\right]} \end{array}$$

$$\sum_{k}{u_{k}}^{2}E\left[{\hat{\pi }}_{k+}^{*}|\left\{V_{1},V_{2},\ldots ,V_{M}\right\}\right]=\sum_{k}{u_{k}}^{2}E\left[\sum_{g}{\hat{\pi }}_{\mathrm{kg}}^{*}|\left\{V_{1},V_{2},\ldots ,V_{M}\right\}\right]$$

$$=\sum_{k}\sum_{g}{u_{k}}^{2}E\left[{\hat{\pi }}_{\mathrm{kg}}^{*}|\left\{V_{1},V_{2},\ldots ,V_{M}\right\}\right]$$

$$=\sum_{k}\sum_{g}{u_{k}}^{2}E\left[\left(\frac{n_{\mathrm{kg}}^{*}}{M}\right)|\left\{V_{1},V_{2},\ldots ,V_{M}\right\}\right]$$

$$=\left(\frac{1}{M}\right)\sum_{k}\sum_{g}{u_{k}}^{2}E\left[\sum_{i}I\left(X_{i}^{*}=k,Y_{i}^{*}=g\right)|\left\{V_{1},V_{2},\ldots ,V_{M}\right\}\right]$$

$$=\left(\frac{1}{M}\right)\sum_{i}\sum_{k}\sum_{g}{u_{k}}^{2}E\left[I\left(X_{i}^{*}=k,Y_{i}^{*}=g\right)|\left\{V_{1},V_{2},\ldots ,V_{M}\right\}\right]$$

$$=\left(\frac{1}{M}\right)\sum_{i}\sum_{k}\sum_{g}{u_{k}}^{2}{\hat{\pi }}_{\mathrm{ikg}}$$

(A5)
$$=\left(\frac{1}{M}\right)\sum_{i}\sum_{k}{u_{k}}^{2}{\hat{\pi }}_{\mathrm{ik}+}$$

Then, $\begin{array}{c} E\left[{\bar{u}}^{*2}|\left\{V_{1},V_{2},\ldots ,V_{M}\right\}\right]=\mathrm{var}\left[{\bar{u}}^{*}|\left\{V_{1},V_{2},\ldots ,V_{M}\right\}\right] \end{array}$ $\begin{array}{c} +{\left[E\left[{\bar{u}}^{*}|\left\{V_{1},V_{2},\ldots ,V_{M}\right\}\right]\right]}^{2} \end{array}$

$$\mathrm{var}\left[{\bar{u}}^{*}|\left\{V_{1},V_{2},\ldots ,V_{M}\right\}\right]=\mathrm{var}\left[\sum_{k}u_{k}{\hat{\pi }}_{k+}^{*}|\left\{V_{1},V_{2},\ldots ,V_{M}\right\}\right]$$

(A6)
$$=\frac{1}{M^{2}}\sum_{i}\sum_{k}{u_{k}}^{2}{\hat{\pi }}_{\mathrm{ik}+}-\frac{1}{M^{2}}\sum_{i}\sum_{k}\sum_{g}{u_{k}}^{2}{\left({\hat{\pi }}_{\mathrm{ikg}}\right)}^{2}$$

(A7)
$$\begin{array}{c} {\left[E\left[{\bar{u}}^{*}|\left\{V_{1},V_{2},\ldots ,V_{M}\right\}\right]\right]}^{2}={\left[E\left[\sum_{k}u_{k}{\hat{\pi }}_{k+}^{*}|\left\{V_{1},V_{2},\ldots ,V_{M}\right\}\right]\right]}^{2}\\ \;=\frac{1}{M^{2}}{\left[\sum_{i}\sum_{k}u_{k}{\hat{\pi }}_{\mathrm{ik}+}\right]}^{2} \end{array}$$

Then, using ((A5), (A6), (A7)), we can obtain the value of (A4) as follows:

$$\begin{array}{c} \sqrt{\left(\frac{1}{M}\right)\sum_{i}\sum_{k}u_{k}^{2}{\hat{\pi }}_{\mathrm{ik}+}-\left(\frac{1}{M^{2}}\right)\sum_{i}\sum_{k}u_{k}^{2}{\hat{\pi }}_{\mathrm{ik}+}}\\ \sqrt{+\left(\frac{1}{M^{2}}\right)\sum_{i}\sum_{k}\sum_{g}u_{k}^{2}{{\hat{\pi }}_{\mathrm{ikg}}}^{2}-{\left[\left(\frac{1}{M}\right)\sum_{i}\sum_{k}u_{k}{\hat{\pi }}_{\mathrm{ik}+}\right]}^{2}} \end{array}$$

Similarly, $\sqrt{E\left[\left(\sum_{g}{v_{g}}^{2}{\hat{\pi }}_{+g}^{*}-{\bar{v}}^{*2}\right)|\left\{V_{1},V_{2},\ldots ,V_{M}\right\}\right]}$ can be expressed as

$$\begin{array}{c} \sqrt{\left(\frac{1}{M}\right)\sum_{i}\sum_{g}v_{g}^{2}{\hat{\pi }}_{i+g}-\left(\frac{1}{M^{2}}\right)\sum_{i}\sum_{g}v_{g}^{2}{\hat{\pi }}_{i+g}+}\\ \sqrt{\left(\frac{1}{M^{2}}\right)\sum_{i}\sum_{k}\sum_{g}v_{g}^{2}{{\hat{\pi }}_{\mathrm{ikg}}}^{2}-{\left[\left(\frac{1}{M}\right)\sum_{i}\sum_{g}v_{g}{\hat{\pi }}_{i+g}\right]}^{2}} \end{array}$$

Finally, we get

$${\hat{\rho }}_{c}==\frac{C_{u,v}}{\sqrt{S_{u}}.\sqrt{S_{v}}}$$

where

$$C_{u,v}=\frac{1}{M}\left(\sum_{i=1}^{M}\sum_{k=1}^{K}\sum_{g=1}^{G}u_{k}v_{g}{\hat{\pi }}_{\mathrm{ikg}}\right)-\frac{1}{M^{2}}\left(\left(\sum_{i=1}^{M}\sum_{k=1}^{K}u_{k}{\hat{\pi }}_{\mathrm{ik}+}\right)\left(\sum_{i=1}^{M}\sum_{g=1}^{G}v_{g}{\hat{\pi }}_{i+g}\right)\right),$$

$$\begin{array}{c} S_{u}=\frac{M-1}{M^{2}}\left(\sum_{i=1}^{M}\sum_{k=1}^{K}{u_{k}}^{2}{\hat{\pi }}_{\mathrm{ik}+}\right)+\frac{1}{M^{2}}\left(\sum_{i=1}^{M}\sum_{k=1}^{K}\sum_{g=1}^{G}u_{k}^{2}{{\hat{\pi }}_{\mathrm{ikg}}}^{2}\right)\\ \;-{\left(\frac{1}{M}\sum_{i=1}^{M}\sum_{k=1}^{K}u_{k}{\hat{\pi }}_{\mathrm{ik}+}\right)}^{2} \end{array}$$

$$\begin{array}{c} S_{v}=\frac{M-1}{M^{2}}\left(\sum_{i=1}^{M}\sum_{g=1}^{G}v_{g}^{2}{\hat{\pi }}_{i+g}\right)+\frac{1}{M^{2}}\left(\sum_{i=1}^{M}\sum_{k=1}^{K}\sum_{g=1}^{G}v_{g}^{2}{{\hat{\pi }}_{\mathrm{ikg}}}^{2}\right)\\ \;-{\left(\frac{1}{M}\sum_{i=1}^{M}\sum_{g=1}^{G}v_{g}{\hat{\pi }}_{i+g}\right)}^{2} \end{array}$$
