# Supplementary material for: An Association Test for Ordinal Outcomes in Clustered Data With Informative Cluster Size
Source: Pharm Stat. 2026 Mar 16;25(2):e70089. doi: 10.1002/pst.70089 (PMC12990042; doi:10.1002/pst.70089)
Supplement: Supplementary file 1 — Table S1: Average bias (Monte‐Carlo based) of the proposed ordinal association estimator ρ^c, under true H0 of no association, for different choices for 𝑀 and the combinations of 𝐾×𝐺 contingency table under the simulation from Section 3.1. Table S2: Average jackknife estimate of the bias of the proposed ordinal association estimator ρ^c, under true H0 of no association, for different choices for 𝑀 and the combinations of 𝐾×𝐺 contingency table under the simulation from Section 3.1. [file PST-25-0-s001.pdf]

Supplementary Web Material

Table S1. Average bias (Monte-Carlo based) of the proposed ordinal association estimator $\hat{\rho}_c$, under true H_0 of no association, for different choices for M and the combinations of $K \times G$ contingency table under the simulation from section 3.1.

Number of clusters (M)	$K \times G$					
	2×2	2×3	2×4	3×3	3×4	4×4
25	0.00067	0.00123	0.00116	0.00188	0.00162	0.00126
50	-0.00112	-0.00071	-0.00113	-0.00056	-0.00100	-0.00092
100	0.00023	0.00058	0.00038	0.00021	0.00032	0.00038

Table S2. Average jackknife estimate of the bias of the proposed ordinal association estimator $\hat{\rho}_c$, under true H_0 of no association, for different choices for M and the combinations of $K \times G$ contingency table under the simulation from section 3.1.

Number of clusters (M)	$K \times G$					
	2×2	2×3	2×4	3×3	3×4	4×4
25	0.00021	0.00034	0.00034	0.00046	0.00038	0.00025
50	-0.00015	-0.00001	-0.00016	-0.00006	-0.00011	-0.00010
100	0.00001	0.00004	0.00003	0.00001	0.00002	0.00002

From the above two tables of simulation results, we can observe that the magnitude of the average bias of the proposed estimator $\hat{\rho}_c$, along with the jackknife bias estimate of $\hat{\rho}_c$, decreases with an increase in M and eventually approaches zero. Therefore, our proposed estimator $\hat{\rho}_c$ becomes approximately, and asymptotically, unbiased for the true association measure under H_0 as the number of clusters (M) increases.
